# Hospital Reports and National Insurance

**Published:** 1913-03-08

**Authors:** 


### HOSPITAL REPORTS AND NATIONAL INSURANCE.

### Newcastle, Sheffield, and West Ham.

The reports of the more important hospitals up and down the country are just beginning to come to hand. Although it is yet too early to form any complete estimate of the effects of National Insurance upon the voluntary hospitals, the reports we have already received indicate the anxiety with which this new legislation is regarded by hospital managers throughout the country.

### NEWCASTLE-ON-TYNE—THE ROYAL VICTORIA INFIRMARY.

At the Royal Victoria Infirmary the house committee are not prepared to commit themselves to any opinion at the present moment as to whether the continuous increase of late upon the beds can or cannot be reduced. Great uncertainty prevails with regard to the effect of the National Insurance Act, and the work of the patients admissions committee is not yet complete. The work of reorganisation has made rapid progress, and the new admission block, costing upwards of £5,000, approaches completion. The Royal Victoria Infirmary contains 430 beds, of which on an average 414 are daily occupied. The margin of vacant beds naturally gives rise to continual anxiety, for although the pressure on the medical side is not so great as that on the surgical, yet it is doubtful whether the accommodation, despite some slight readjustment of beds, will be found to be sufficient for the next five years. The tendency of the National Insurance Act must be to increase the number of applicants for in-patient treatment, and this extraordinary pressure may soon cause the present number of beds to be insufficient to meet the demand. As the hospital is being worked to its utmost capacity at present, very careful investigation is being instituted into the circumstances of all applicants for treatment with a view to confine admissions to suitable cases only. Apart from the effects of the National Insurance Act, there is no reason to anticipate more than the inevitable increases in annual expenditure due to new fashions in treatment, increased prices, and the continuous growth in personnel.

The Royal Victoria Infirmary occupies a very prominent position in the eyes of the hospital world at the present time from the circumstance that, whilst it has the most democratic form of government of any hospital in the world, it is still able to retain its voluntary character. The fact that the support of the working classes to this hospital yields a revenus of £20,000 a year indicates that modifications will have to be introduced into our hospital system and practically a new kind of voluntary hospital evolved which will not be exactly upon the old lines, but will be so organised and administered as to retain all the best features of the voluntary principle and to protect it from the dangers and evils which have arisen in State hospitals in this and other countries. The total ordinary income in 1912 was £31,755, of which some £18,700 was derived from workmen's contributions. Legacies in 1912 yielded £14,759, and the total income from all sources was £46,514, against a total expenditure under all heads of £41,000.

Seeing that the working men contribute considerably more than half the ordinary income of this institution, it is but right that they should be well represented on the house committee. The working men at the commencement of 1912 had sixteen representatives thereon, which is probably a larger proportion than that held by artisan classes in connection with any voluntary hospital. It would appear, however, as if the working men of Newcastle and the district, which includes the miners of Northumberland, Durham, North and South Tyne, Newcastle, Gateshead and Blaydon districts, and of Elswick, have insisted upon having a larger representation, for at the November quarterly court the number of working men representatives on the house committee was increased by 50 per cent., or from sixteen to twenty-four members. In one sense, in view of the threatened income, it is encouraging to note that the artisans who at present support the Royal Victoria Infirmary are thoroughly alive to its value, and that they could, we are credibly informed, without very much difficulty, double the amount of support at present given to it if they wished to do so.

So far as the support of other classes of the community is concerned the legacies for 1912 were above the average, though the bequests received were no doubt included in wills made several years ago. A few employers of labour have either withdrawn their subscription or have expressed their intention of doing so on account of recent legislation, but it is too early to conclude with fairness that this attitude is likely to become general and permanent. One of the most encouraging and interesting features to note is the fact that a lady almoner has recently been appointed and is now working in co-operation with the other charitable agencies in Newcastle and the district. Her duties will be to fulfil the double purpose of helping to check hospital abuse, whilst she will endeavour to the utmost to be really helpful to the patients. It is the lady almoner's especial care to look after many patients who are unable from the poverty of their home surroundings to make the best of the advice and help which the infirmary may give. Another important departure is the appointment for the first time of a resident medical officer through whose hands all patients will be admitted to the infirmary. This institution, under the able superintendence of Mr. Roden Orde, with its active and strong committees, presents much that is full of interest and importance to hospital managers everywhere.

### SHEFFIELD—THE ROYAL INFIRMARY.

The Royal Infirmary is the most important voluntary institution in Sheffield, and as we have had occasion to state, has well maintained its splendid traditions with increased efficiency during recent years. Seven years ago it contained 247 beds with 3,354 in-patients; at the end of 1912 the beds amounted to 326 with 3,913 in-patients. During these seven years the out-patients have increased by more than 5,000, the casualties by over 4,000, the annual income has increased by £1,400, and the ordinary expenditure by £4,000, figures which reveal causes which must give anxiety to the managers. Again, whilst the voluntary subscriptions in seven years show a small increase of nearly £200 and the workmen's contributions one of about £700, the amount derived from legacies has fallen from £9,516 in 1906, to £2,299 in 1912. The reason for the increased expenditure, and inferentially for the increase in the number of in-patients, at any rate, may be traced to a decision of the board given years ago, to erect a new ward block to be devoted solely to surgical cases, and to reconstruct some of the older portions of the existing hospital buildings for the accommodation of the increased staff of nurses and servants, and for administrative purposes. This new block came into full occupation in 1912, and contains accommodation for nearly 200 patients. In consequence, the laundry had to be considerably enlarged, and new boiler houses erected to supply the whole of the heating of the infirmary, together with hot water for the steam cooking, domestic, and other purposes. Although the contributions of the citizens of Sheffield were liberal, there is still a sum of £12,000 owing on these extensions and alterations, which we hope will be wiped off before the end of the current year.

We have said already that the outlook for the managers, financially, is an anxious one. They feel that the future of hospitals generally, and of this infirmary, cannot be viewed with any degree of optimism. Recent legislation has been a severe blow to voluntary hospitals, and this Infirmary has to face the loss of forty-one annual subscribers who discontinued their subscriptions during 1912 on account of the National Insurance Act. Nearly every one of these were approached and urged to continue, but they have remained obdurate. So an active canvass has had to be set on foot for new subscriptions, which has been helped by the increased prosperity of the city at the moment, and an energetic collection, with satisfactory results. The managers fear, however, that a decided decline in trade must mean a very considerable falling-off in subscriptions, both from employers and workmen. It is held in Sheffield, we understand, that the wells of charity are being slowly dried up through recent legislation, a fact which is eloquently testified to by the drop in legacies during the past six years. The figures are: 1906, £9,516; 1907, £5,385; 1908, £3,778; 1909, £3,845; 1910, £5,020; 1911, £1,196. One remarkable feature in the experience of the Royal Infirmary, Sheffield, is that during a recent fortnight there were admitted to this hospital 64 patients who had no dependants, so that Section 12 of the National Insurance Act, if the Committee promptly enter into an agreement with the Insurance Committee and Approved Societies, may be made to yield a substantial sum by the payment of the whole, or part of, the sickness benefits to the hospitals, instead of to the sixty-four patients in question. There is a strong feeling of injustice at the Chancellor of the Exchequer's decision to refuse the voluntary hospitals relief from legacy duty, and the hospital's supporters regard this act on Mr. Lloyd George's part as proving a lack of sympathy towards voluntary hospitals by the present Ministers of his Majesty's Government.

### THE WEST HAM AND EASTERN GENERAL HOSPITAL.

The authorities of this hospital have some fear that there may be a reduction of subscriptions and donations owing to the Insurance Act. The energetic chairman, Mr. Alderman Cook, stands stoutly by the view that the need for the hospital to the residents in the district which it serves will make itself so felt as to yield an increased number of subscribers. This hospital, unlike the two provincial hospitals just referred to, though situated in the centre of a working-class area, receives but small support from the working men. Thus, though the expenditure in 1912 was over £11,000, the workmen's donations amounted to only £314. During the last ten years this institution has been so enlarged and improved at a cost of £40,622 as to give it a new importance to the district which it serves. The additions to the new buildings have provided accommodation for forty additional beds for sisters and nurses, a new operation theatre unit, new administration offices, an out-patient department, new children's wards, a chemical laboratory, dispensary, and stores, new quarters for the resident medical and domestic staff, a pathological block, laundry additions, balconies, and an installation of electric light throughout the hospital. Between 1902 and 1912 the annual subscriptions have increased from £1,100 to nearly £1,500; the workmen's donations decreased from £363 to £314; the donations have been well maintained, and, including the annual dinner, reached a total of £5,120. The in-patients have increased from 810 to 1,521 per annum, the casualties from 9,500 to 20,187, and the total out-patients from 23,000 to 39,000. It will be observed that there has been an enormous increase in casualties, and the total attendances have grown from 83,000 to 128,000. Attendances have, however, been materially reduced in numbers owing to the appointment of a lady almoner who was at work throughout 1912. We congratulate Alderman Cook upon the marked and increasing success which has attended the self-denying labours he has expended upon this institution for eleven years past.

